# Ingestion of an Amino Acid Electrolyte Beverage during Resistance Exercise Does Not Impact Fluid Shifts into Muscle or Performance

**DOI:** 10.3390/sports5020036

**Published:** 2017-06-10

**Authors:** JohnEric W. Smith, Ben M. Krings, Timothy J. Peterson, Jaden A. Rountree, Roksana B. Zak, Matthew J. McAllister

**Affiliations:** 1 Department of Kinesiology, Mississippi State University, Mississippi State, MS 39762, USA; bmk216@msstate.edu (B.M.K.); tjp91@msstate.edu (T.J.P.); jarountree@crimson.ua.edu (J.A.R.); mjm639@msstate.edu (M.J.M.); 2 School of Health and Kinesiology, University of Nebraska-Omaha, Omaha, NE 68182, USA; rzak@unomaha.edu

**Keywords:** supplementation, muscle pump, muscle fatigue, ergogenic aid

## Abstract

The purpose of this study was to investigate the impact of ingesting an amino acid-electrolyte (AAE) beverage during upper body resistance exercise on transient muscle hypertrophy, exercise performance, markers of muscle damage, and recovery. Participants (*n* = 15) performed three sets of six repetitions—bench press, lat pull down, incline press, and seated row—followed by three sets of eight repetitions at 75% of the estimated 1 repetition maximum—triceps kickback, hammer curl, triceps push down, and preacher curl—with 90 s of rest between sets. The final set of the push down/preacher curl was performed to failure. Prior to and immediately post-exercise, as well as 24, 48, and 72 h post exercise, cross-sectional muscle thickness was measured. Blood samples were collected prior to exercise, as well as 24, 48, and 72 h post-exercise for serum creatine kinase (CK) analysis. No treatment effect was found for muscle cross-sectional area, repetitions to failure, or serum CK. A main effect (*p* < 0.001) was observed in the change in serum CK levels in the days following the resistance exercise session. The findings of this study suggest that the acute ingestion of a AAE beverage does not alter acute muscle thickness, performance, perceived soreness and weakness, or markers of muscle damage.

## 1. Introduction

Maintaining appropriate hydration status at moments of activity is encouraged by most athlete influencers and sports science organizations. Current fluid recommendations, such as those given by the American College of Sports Medicine [1], are primarily developed in prolonged endurance training and press the need for fluid and electrolytes to promote fluid absorption, with the allowance for a small amount (6%–8% total volume) of carbohydrate. However, previous research has provided a criticism that amino acid and protein ingestion does not provide additional performance benefits during exercise due to increased time needed for absorption [2,3]. Increases in time needed for gastric emptying resulting from protein or amino acids may also slow delivery of fluid or other nutrients (i.e., carbohydrates) contained in the supplemented food or beverage.

While there are potential negative effects to ingesting amino acids and protein during exercise in terms of hydration, resistance-trained athletes often incorporate dietary amino acid supplements to possibly enhance exercise performance, minimize muscle damage, and facilitate muscle repair from training [4,5]. Previous research has reported that branched-chain amino acid (BCAA) supplementation during resistance exercise, as well as endurance exercise, may attenuate indirect markers of skeletal muscle damage, such as the enzyme creatine kinase (CK) [6,7,8]. In addition, BCAA supplementation prior to and during resistance exercise has been shown to significantly decrease serum cortisol when compared to a placebo control [8]. These responses may indicate an anticatabolic hormonal effect when ingesting BCAA before and during resistance exercise, and may attenuate muscle protein breakdown [5,8]. Given the high potential for variability in CK levels, serum CK concentrations before, during, and after a resistance exercise protocol may provide an indication of overall muscle damage throughout the processes of training and recovery [9]. 

The absorption of fluid and nutrients contained within a beverage could be a limiting step in the efficacy of a sports hydration/nutrition product. It could be theorized that changes in fluid absorption could inhibit some of the fluid shift into the exercising muscles typically seen during exercise as fluid shifts into muscle. Taking into consideration the desire of resistance trained athletes to ingest amino acids in an effort to improve muscle recovery and maintain a positive protein balance, the purpose of this study was to investigate the impact of an amino acid-electrolyte (AAE) beverage during an upper body resistance exercise (RE) protocol on fluid shift into the muscle (as assessed by cross-sectional muscle thickness), RE performance, markers of muscle damage, and recovery. It was hypothesized that ingestion of an AAE beverage during RE sessions would not negatively impact fluid shifts into the muscle and would result in improved performance, decreased biological markers of muscle damage, and reduced muscle soreness as compared to the ingestion of an electrolyte-only placebo beverage.

## 2. Methods

### 2.1. Participants

Eighteen healthy resistance-trained males (23.36 ± 3.42 years, 177.44 ± 5.98 cm, 82.73 ± 9.43 kg) provided written informed consent prior to participation in the study. Participants were required to have participated in RE for 2–3 days per week for at least 6 months prior to the start of the study. Participants were instructed to refrain from supplementation beginning two weeks prior to the initial trial and participation in any additional RE throughout the duration of the study. Data from three participants were not included due to non-adherence to exercise restrictions during the study, resulting in only 15 participants being analyzed. 

### 2.2. Experimental Design

Prior to any testing, all procedures were approved by the Mississippi State University Institutional Review Board. This study utilized a randomized, double-blind, placebo-controlled, within-participant’s crossover design. The participants were randomly assigned to treatments and ingested either an amino acid-electrolyte (AAE) beverage or an electrolyte-only placebo (PLA) control beverage before and during two acute RE sessions. RE consisted of both bilateral and unilateral exercises for which participants were randomly assigned to dominant vs. non-dominant unilateral performance assessments. To analyze the relationships between amino acid supplementation and characteristics of muscle damage, participants’ blood samples were analyzed for serum CK levels. Muscle cross-sectional thickness, along with perceived soreness and weakness was assessed before, during, and after acute RE protocol.

### 2.3. Experimental Protocol

#### 2.3.1. Baseline Testing 

Participants reported to the training facility (Sanderson Center, Mississippi State, MS, USA) one week before experimental trials for one repetition maximum (1 RM) estimations. The 1 RM assessment consisted of a warm-up on the treadmill for 5 min at 5 mph and 0% grade and eight upper body exercises. These exercises included bench press, lat pull down, incline press, seated row, triceps kickback, hammer curl, triceps pushdown, and preacher curl—performed in this order. Each exercise consisted of a warm-up set and a maximal repetition set with 90 s of rest between sets. For each warm-up set, participants self-selected weights that they could lift for 20 repetitions, but were stopped at 10 repetitions by research personnel to standardize training volume. For the max sets, participants selected weights that they could lift more than one but no more than 10 times and performed as many repetitions as they could until failure. Estimated 1 RMs were determined by the following formula [10]:

$$\text{Estimated }1\text{ RM }\left(kg\right)=\frac{\mathrm{weight}\;\mathrm{lifted}\;\left(\mathrm{kg}\right)}{\left[100-\left(\mathrm{reps}\cdot 2.5\right)\right]÷100}$$

Participants reported to the laboratory 24 h prior to the experimental trial after an overnight 10 h fast for baseline assessments of perceived muscle soreness and weakness, cross-sectional muscle thickness of left and right biceps brachii and triceps brachii, and blood draw. A visual analog scale (VAS) was used to determine perceived muscle soreness and weakness of the left and right biceps and triceps. 

Muscle cross section area (CSA), as an assessment of transient muscle hypertrophy, was assessed using a LOGIQ e Diagnostic Ultrasound System (General Electric, Wauwatosa, WI, USA). An ultrasound transducer with a bandwidth of 5.0–13.0 MHz imaging frequency and a 12.7 × 53 mm footprint was used to take all muscle CSA measurements (General Electric, Wauwatosa, WI, USA). CSA measurements were taken immediately prior to, as well as post exercise, with follow-up measurements conducted at 24 and 48 h post exercise as well. Two measurements were taken for each muscle, and the average of the two was subsequently used for analysis. The participants were in a seated position for each assessment with their arms rested at their side (elbows extended) and muscles were relaxed. Measurements were taken 1 to 2 min after participants assumed the testing position. The ultrasound measurement location for the triceps brachii was standardized at 60% of the measured distance from the acromion process to the olecranon process along the muscle belly, and horizontally from that point to the muscle belly of the biceps brachii. Placement of the ultrasound transducer head was marked with a permanent marker to ensure consistent placement of the ultrasound probe for the subsequent measurements. The distance (cm) from the humerus to the edge of the muscle belly constitutes the CSA. The same technician conducted all CSA assessments to maintain consistency between trials.

#### 2.3.2. Experimental Trials

Experimental trials consisted of two RE sessions, separated by one week. Given that our participants were experienced with RT, we expected the repeated bout effect to be minimal [11]. However, in an attempt to avoid the repeated bout effect, unilateral exercises were performed and the opposite side was trained on alternating testing sessions. Perceived soreness and weakness along with cross-sectional muscle thickness were measured before and immediately after training, as well as 24, 48, and 72 h post-training. CK was measured 24 h prior to the training session, as well as 24, 48, and 72 h post-training. 

Participants reported to the training facility after an overnight 10 h fast during RE sessions. During both RE sessions, participants ingested 237 mL every 15 min (948 mL during the trial) of either an AAE or electrolyte only PLA. The differences between the two treatments were 0.67 g L-Leucine, 0.32 g L-Isoleucine, 0.32 g L-Valine, 1 g L-Taurine, 0.5 g L-Citrulline delivered per serving of the AAE beverage. AAE and PLA beverages both contained 57.5 mg Na^+^, 32.5 mg K^+^, 10 mg Ca^++^, and 6 mg Mg^+^ per serving. Both treatments were similar in taste, appearance, and scent. All treatments were manufactured by Dymatize Enterprises, LLC. (Dallas, TX, USA) for this study. The first round of ingestion occurred immediately before commencing the RE protocol. The RE protocol included a treadmill warm-up (5 min at 5 mph and 0% grade) and eight exercises (four bilateral: bench press, lat pull down, incline press, seated row, followed by four unilateral: triceps kickback, hammer curl, triceps push down, preacher curl). Participants were monitored by personnel with the NSCA CSCS certification while they performed three sets of six repetitions for the bilateral exercises and three sets of eight repetitions at 75% of the estimated 1 RM for the unilateral exercises with 90 s of rest between sets. The last two unilateral exercises (triceps push down, preacher curl) were performed as a superset. During the third superset, participants performed repetitions until muscular failure without encouragement or feedback from researchers. Immediately after the final superset, CSA was measured in addition to the recording of perceived soreness and weakness. Participants reported to the laboratory 24, 48, and 72 h after the training session for assessment of perceived soreness and weakness, CSA, and CK.

### 2.4. Blood Analysis

Blood (7 mL) was drawn from an antecubital vein into a non-treated vacutainer. The blood was allowed to clot for 30 min at room temperature and then centrifuged for 10 min at 2500 rpm and 4 °C. Serum was aliquoted and stored at −40 °C until sample analysis. Each sample was analyzed for CK in duplicate using a Pointe Scientific Pointe 180 II Spectrophotometer (Ann Arbor, MI, USA), according to specific kinetic assay procedures. An amount of 25 μL of serum was analyzed in 1 mL of CK reagent at room temperature.

### 2.5. Statistical Analysis

Data are presented as mean ± standard deviation. A priori significance was set at *p* < 0.05. Data were analyzed using SPSS version 21 (Armonk, NY, USA). Work was analyzed using a paired sample *t*-test. Changes between treatments and days in CSA, soreness and weakness, and CK were analyzed using a two-way repeated measures analysis of variance (time × trial). Duncan post-hoc test was used when appropriate [12].

## 3. Results

**Figure 1:**
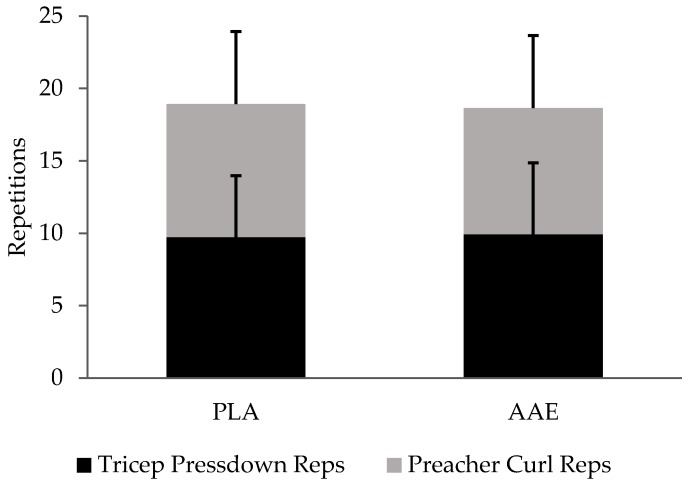
Impact of an amino acid-electrolyte (AAE) beverage on muscle fatigue during superset repetitions to muscular failure. Data are presented as mean ± SD.

There was no difference found in the total work completed between treatments when comparing repetitions completed in the final super set of triceps push down and preacher curls (*p* = 0.80) (See Figure 1). 

**Figure 2:**
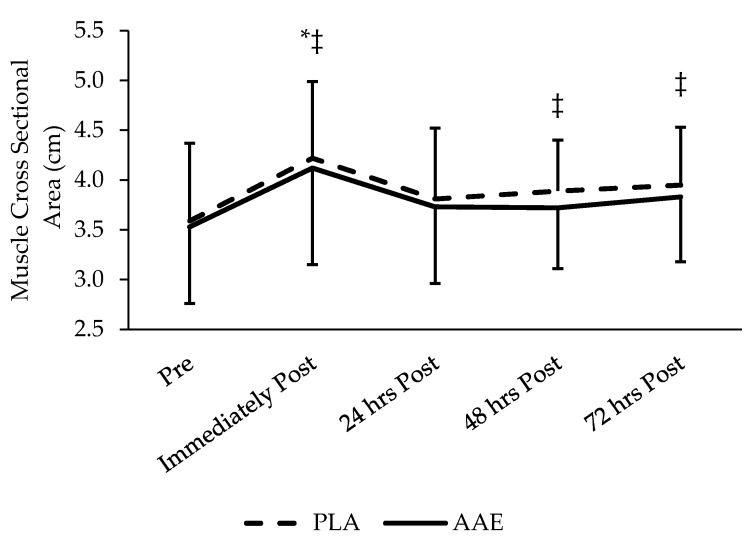
Impact of an AAE beverage on muscle thickness in the 3 days following resistance exercise with acute supplementation. No difference between treatments. ‡ Denotes significant time difference from pre (*p* < 0.05). * Denotes significant time difference from 24 h post, 48 h post, and 72 h post. Data are presented as mean ± SD.

No significant differences were found regarding changes in muscle CSA (Figure 2) with the AAE during RE compared to PLA (*p* = 0.49). 

**Figure 3:**
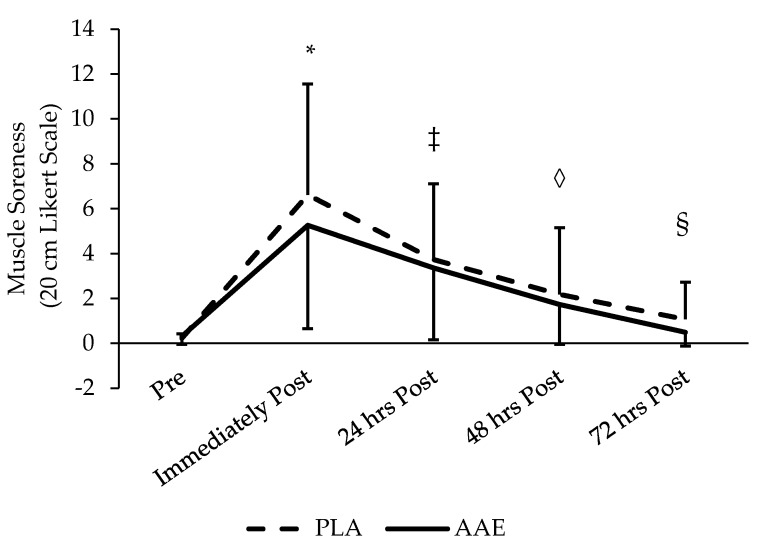
Impact of AAE beverage on muscle soreness in the 3 days following resistance exercise with acute supplementation. No significant difference between treatments. *, ‡, ◊, and § used to identify all time points different from one another (*p* < 0.05). Data are presented as mean ± SD.

**Figure 4:**
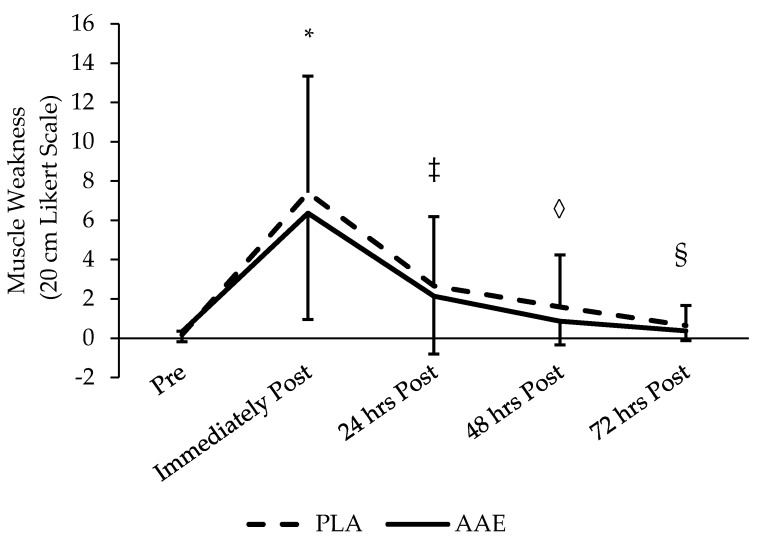
Impact of an AAE beverage on muscle weakness in the 3 days following resistance exercise with acute supplementation. No difference between treatments. *, ‡, ◊, and § used to identify all time points significantly different from one another (*p* < 0.05).

No treatment effect was found for perceived muscle soreness or weakness measured by VAS (*p* = 0.17 and *p* = 0.29, respectively) (Figure 3 and Figure 4). However, a significant main effect was found for soreness and weakness between days (*p* < 0.001) indicating that the training protocol was effective at eliciting significant changes in perceived muscle soreness and weakness. Soreness and weakness was highest immediately after the training session, but there was no significant increase. Soreness and weakness declined significantly each day until returning to baseline 48 h after training. 

**Figure 5:**
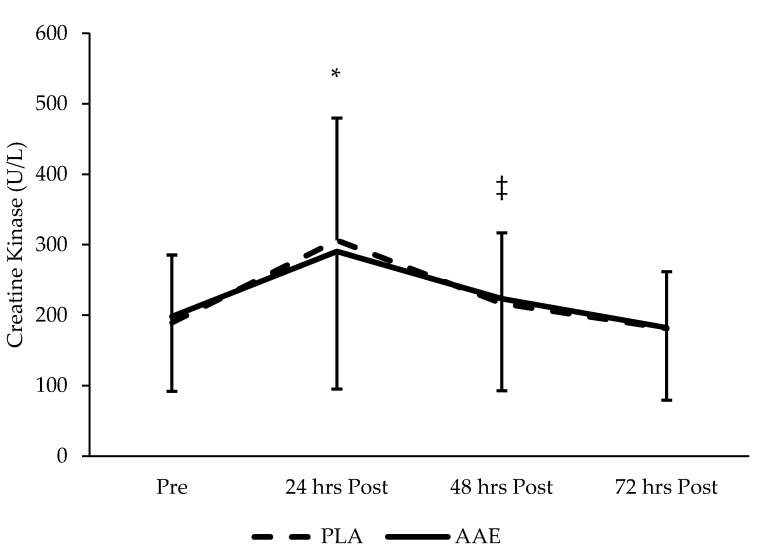
Impact of an AAE beverage on serum CK in the 3 days following resistance exercise with acute supplementation. No difference between treatments. * Denotes significant time difference (*p* < 0.05) from pre, 48 h post, and 72 h post. ‡ Denotes significant difference from 72 h post. Data are presented as mean ± SD.

The changes in CK levels between days and treatments are shown in Figure 5. Mean CK levels were not significantly different between treatments (*p* = 0.99). A significant main effect (*p* < 0.001) was observed in the change in serum CK levels over the course of the days following the RE session. The exercise protocol elicited a significant increase in serum CK levels (*p* < 0.001). The change in serum CK levels as compared to the baseline measure was significantly greater 24 h after training, but otherwise returned to resting levels.

## 4. Discussion

As hypothesized, ingestion of an AAE beverage did not negatively affect transient muscle hypertrophy, as assessed by muscle cross-sectional area. The hypothesis that an AAE beverage would improve performance, minimize muscle damage, and promote muscle recovery in response to acute RE was not supported with the findings of this study. However, others have demonstrated that the ingestion of an AAE beverage during exercise may result in enhanced muscle recovery since isolated essential amino acids and protein have been shown to enhance protein synthesis [13,14,15]. 

Studies suggesting the potential for protein and amino acids to decrease absorption and fluid absorption have often examined beverages with protein and amino acids added in addition to carbohydrate [3]. While total body water was not assessed in this study, the practical fluid shifts into the muscle were estimated using muscle CSA. The amino acid content of the beverage investigated in this study provided four servings of 0.67 g L-Leucine, 0.32 g L-Isoleucine, 0.32 g L-Valine, 1 g L-Taurine, and 0.5 g L-Citrulline in addition to electrolytes. This small amount of amino acids may not have caused significant changes in absorption or intestinal absorption due to their relatively minor impact on beverage osmolality as compared to carbohydrate + protein beverages. 

Calorically, the AAE beverage ingested during each training session contained approximately 44 kcals. Even with carbohydrate, a major exogenous fuel source during exercise [16], low caloric intakes during exercise provide only limited performance changes [17]. Essential amino acids are not a preferred substrate for cellular energy production, therefore it is likely that the addition of carbohydrates to the AAE beverage may acutely improve performance. For these reasons, the lack of significant change in acute performance being found between the AAE beverage and PLA is not surprising. The addition of a more predominant energy substrate such as simple carbohydrates in combination with AAE would likely transition to acute increases in performance [18]. BCAA ingestion before and during exercise has been shown to reduce the ratings of perceived exertion during endurance exercise [19,20,21], improve recovery from exercise [4,5], and increase protein synthesis with or without the addition of carbohydrates [22,23,24]. However, care must be taken as the finding of enhanced protein synthesis after exercise lacks consistency [25].

Serum CK values in relation to baseline measures increased 24 h after exercise and demonstrated a decline in concentration lower than baseline measures. This negative change is likely the result of the elevated serum CK values measured at baseline. All participants avoided training for at least 72 h prior to the baseline, however, resting serum CK levels may be elevated 4–5 days post-training [9]. The RE protocol elicited a significant elevation in serum CK levels which was similarly reported by previous investigations [26,27]. 

Our study is not without limitations. We advised participants to maintain similar dietary habits for the 24 h leading up to each experimental session. We recognize that not recording dietary information and specifically, dietary protein intake, is a limitation of the current study. However, since the primary goal of the study was to observe fluid shifts during upper body RE, the authors did not believe that chronic dietary protein ingestion affected acute fluid shifts into muscle. Elevations in CK levels prior to baseline could be a limitation of this study since the participants practiced habitual resistance exercise. 

Statistically, the ingestion of an AAE beverage did not provide beneficial effects compared to PLA. Future research should investigate the effects of longitudinal ingestion of an AAE beverage during resistance training sessions. It is possible that AAE ingestion during resistance exercise may not provide a significant increase in performance or decrease in muscle soreness and weakness, but athletes often experience noticeable improvements in their performance. The effect needed to experience a meaningful performance improvement is valuable to an athlete even without statistical benefit [28]. 

## 5. Conclusions

Collectively, these data demonstrate no direct benefit or detriment of ingesting AAE beverages on exercise performance, fluid shifts into muscle, perceived muscle soreness and weakness, and serum CK levels over a non-caloric placebo. However, future studies should investigate the potential long-term effect of AAE beverages in combination with resistance exercise.
